# An SVD-based comparison of nine whole eukaryotic genomes supports a coelomate rather than ecdysozoan lineage

**DOI:** 10.1186/1471-2105-5-204

**Published:** 2004-12-17

**Authors:** Gary W Stuart, Michael W Berry

**Affiliations:** 1 Department of Life Sciences, Indiana State University, Terre Haute, IN 47809, USA; 2 Visiting Scientist, Center for Genomics and Bioinformatics, Indiana University, Bloomington, IN 47405, USA; 3 Department of Computer Science, University of Tennessee, Knoxville TN 37996-3450, USA

## Abstract

**Background:**

Eukaryotic whole genome sequences are accumulating at an impressive rate. Effective methods for comparing multiple whole eukaryotic genomes on a large scale are needed. Most attempted solutions involve the production of large scale alignments, and many of these require a high stringency pre-screen for putative orthologs in order to reduce the effective size of the dataset and provide a reasonably high but unknown fraction of correctly aligned homologous sites for comparison. As an alternative, highly efficient methods that do not require the pre-alignment of operationally defined orthologs are also being explored.

**Results:**

A non-alignment method based on the Singular Value Decomposition (SVD) was used to compare the predicted protein complement of nine whole eukaryotic genomes ranging from yeast to man. This analysis resulted in the simultaneous identification and definition of a large number of well conserved motifs and gene families, and produced a species tree supporting one of two conflicting hypotheses of metazoan relationships.

**Conclusions:**

Our SVD-based analysis of the entire protein complement of nine whole eukaryotic genomes suggests that highly conserved motifs and gene families can be identified and effectively compared in a single coherent definition space for the easy extraction of gene and species trees. While this occurs without the explicit definition of orthologs or homologous sites, the analysis can provide a basis for these definitions.

## Background

Several methods have been developed for the detailed global comparison of multiple whole genomes and the production of global phylogenies. Most of these methods require the prior identification and selection of a reasonably small subset of putative orthologs within which individual homologous sites are identified with some degree of confidence using alignment [1-7]. Frequently, detailed alignment information is subdivided and compressed into a smaller number of complex characters (such as gene content or gene order), which are then used for quantitative comparison [[4,5]; see [6] for review], but the more or less direct use of large scale sequence alignments have also been attempted [7].

Though generally less developed, many non-alignment methods, considered initially by Blaisdell [8], are currently being explored for a similar purpose [[9-15]; see [17] for review]. Rarely do such methods simultaneously provide 1) detailed and unbiased comparisons of a high fraction of biomolecular sequences within full genome datasets, and 2) globally consistent gene and species trees based on this exhaustive comparison. We have recently developed an SVD-based phylogenetic method that provides accurate comparisons of a high fraction of sequences within whole genomes without the prior identification of orthologs or homologous sites [13]. This method has been successfully applied to a number of diverse genome datasets, including mitochondrial genomes, bacterial genomes, and viral genomes [13-15]. Here we apply this method to a diverse set of nine complete eukaryotic nuclear genomes, resulting in the production of a species tree based on the automatic identification and simultaneous comparison of over 400 conserved amino acid motifs and gene families.

## Results and discussion

### Proteome data sets and sequence conversion

The nine eukaryotic genomes compared in this analysis are listed in Table 1. The protein sets obtained from NCBI for the malaria parasite (Pfal) and the budding yeast (Scer) each contributed only 3% of the 175,559 total proteins in the dataset, while the proteins for Frub provided nearly 21% of the total. Only the Frub proteins were obtained from the Ensemble Genome Browser [16], since protein predictions for this organism were not available from NCBI. Differences in methods used to predict proteins by these two organizations might be responsible for the large difference in the number of proteins predicted for comparable vertebrate genomes (>37,000 for Frub, but only 21–25,000 for all other vertebrates). These differences could, in principle, drastically effect the gene and species trees derived from a global comparison of all proteins. However, the position of Frub in the final species tree suggests that these effects were relatively minor (see below). We have noted previously that even drastic genome size differences can be accommodated by our method [14].

### SVD-derived vector definitions for motifs and gene families

All the proteins in the dataset were recoded as overlapping tetrapeptide frequency vectors and the resulting data matrix was decomposed by the SVD. A total of 437 singular triplets were obtained as output. The "protein" vectors provided in the "right" factor matrix are known to provide reduced dimensional definitions for all proteins in the dataset as linear combinations of the orthogonal "right" singular vectors (rsv's). Conversely, the right singular vectors themselves frequently represent "ideal" versions of proteins defining a given gene family [13,14] Protein vectors having the strongest projections on a given rsv are therefore likely to represent members of a given gene family. In this analysis, the proteins with the five strongest projections (referred to as the "top 5") for each rsv were used to identify and summarize a number of gene families. The total number of proteins from each species that appear in the "top 5" for all 437 right singular vectors are listed in Table 1. Although the fraction of "top 5" proteins identified by the SVD roughly parallels the fraction of total proteins from each species, the mammalian proteins tend to dominate the analysis.

Each right singular vector can potentially define two distinct gene families. In this case, the highest positive elemental values within a vector identify proteins associated with one protein family, while the highest negative values identify proteins associated with an anti-correlated family (i.e. proteins that rarely share the same tetrapeptides). Frequently, however, strong family definitions are provided for just one protein family. In this case, the anti-correlated proteins are seen to be derived from a mixture of two or more families. Since the choice of sign is arbitrary, strong family definitions are equally likely to be provided by either the positive or the negative values within a vector. Family definitions provided by positive vector values are denoted below using the simple vector index (e.g. 277 = the 277^th ^singular vector). Those provided by negative vector values are followed by an "a" (e.g. 277a). Its worth noting at this point that protein family definitions provided by the SVD necessarily account for not only "what is there" (tetrapeptides that form the motifs that define the family), but also "what is not there" (tetrapeptides excluded by that family of proteins, but likely to form anti-correlated motifs within other families of proteins).

### Protein family definitions provided by right singular vectors

An abbreviated list of 58 protein families identified within the 437 SVD-derived singular triplets are provided in Table 2. For each listed singular triplet, the gi# of an example protein chosen from among the "top 5" values within the right singular vector is provided, along with its corresponding Name and a Protein Description provided within the NCBI annotation for that protein. In general, proteins described by the more dominant singular triplets were selected for presentation from the complete list of 437 triplets. However, some were chosen due to their historical utility for evolutionary comparisons (ribosomal proteins) and/or their tendency to be accompanied by strongly correlated peptide motifs (last column of Table 2). Relatively few families appear in the table due to the fact that some vectors strongly describe only one family rather than two, some vectors describe only families from species that lack annotation or are poorly annotated at NCBI (i.e. Frub proteins, Agam proteins, etc.), some vectors describe protein families listed by NCBI merely as "unknown" or "conserved unknown", some vectors describe proteins with weakly conserved motifs, and some vectors describe distinct subfamilies of proteins. In the latter case, multiple right singular vectors are apparently required in combination to describe some of the more diverse families of proteins. Included in Table 2 is the number of singular vectors that include the chosen example protein within its "top 5". When multiple vectors are involved in defining multiple related subfamilies, the most "dominant" vector (the one on which the example protein casts its strongest projection) is listed in the first column. Thus, some proteins are seen to have multiple subfamily affiliations. The multiple vectors observed per family effectively subdivide the 58 families into 179 distinct subfamilies. For instance, Table 2 includes a set of 18 ribosomal protein families described by a total of 65 singular vectors (highlighted in bold). Ribosomal proteins are frequently well conserved, effectively aligned, and commonly used for estimating evolutionary relationships. Their presence within our list of dominant singular vectors suggests their utility for establishing evolutionary relationships even in the absence of explicit alignments and explicit *a priori *assignments of orthology.

The diverse families of ras proteins present within the eukaryotic data set provide good examples of the ability of SVD-derived singular triplets to identify and describe both superfamilies and subfamilies of proteins. The ras proteins are well described by at least 13 vectors, including the 6 dominant vectors highlighted in italics in Table 2. All the "top 5" members of the protein families identified by these 6 vectors are listed in Table 3. Vector 197a summarizes the brain-associated ras11 subfamily (Rab11), vector 236a summarizes the Aplysia-related ras subfamily (ApRas), vectors 277 and 277a summarize the brain-associated ras 5 subfamily (Rab5) and the complex Ha/K/Nras subfamily (HaRas) respectively, vector 350a summarizes the ras-related C3 botulinum toxin substrate 1 subfamily (Rac1), and vector 387a summarizes the brain-associated ras1B subfamily (Rab1B). The most dominant ras vector, 389a, appears to describe a more generalized version of the Rab1 subfamily, since this vector includes both Rab1A and Rab1B proteins within the "top five". In addition, as explained below, this vector also summarizes a high fraction of the entire set of 34 ras sequences within all subfamilies.

For comparison, KOG and Homologen memberships are also listed, when available, for each of the "top 5" proteins listed in Table 3. Table 4 provides a similar comparison for a set of four arbitrarily selected protein families unrelated to ras or to each other (potassium channel, enolase, solute carrier protein, and ADP-ribosylation factor). Since most of the genomes used in our study have not yet been included within the KOG classification scheme, only fly and human proteins have official KOG affiliations. However, we expect with high likelihood that most if not all of the top 5 proteins listed in Tables 3 and 4 would also be members of the particular KOG family listed for each vector. Given this, there would be a good correspondence in Tables 3 and 4 between KOG family members and the proteins identified by singular vectors. In contrast, the Homologen resource appears to provide a more selective classification method, dividing the KOG protein families into two or more subfamilies within which members are more likely to represent specific orthologs.

### Conserved motif definitions provided by left singular vectors

Members of any particular ras subfamily represented by a given right singular vector share a uniquely conserved set of correlated tetrapeptides we have previously referred to as a "copep motif". These motifs are explicitly described by the corresponding "left" singular vectors (lsv's) comprising a given singular triplet. The lsv's describe these copep motifs as linear combinations of the 160,000 possible tetrapeptides. Those with high positive values identify peptides found with high probability in the conserved motif of a given subfamily, while those with a high negative value identify peptides excluded with high probability. Therefore, like the rsv's, the lsv's frequently describe two distinct anti-correlated entities (in this case motifs rather than protein families) using either positive or negative values within the vector. Using essentially the same procedure described above for any given rsv, the tetrapeptides having the largest positive or largest negative projections on any given lsv were identified in order to provide a focused summary of the motifs described by that vector. For motif extraction, however, an arbitrary cut-off value (absolute value > 0.025) was used to identify dominant tetrapeptides.

In most cases, it is possible to cluster the resulting short list of dominant tetrapeptides into several uninterrupted copep strings formed by tetrapeptides that overlap in 3 of 4 consecutive amino acid positions. Using this procedure, one long copep string was identified for each of the singular triplets listed in Table 2. The length of the identified long copep string and its corresponding E-value (resulting from pairwise BLAST) are provided as a summary in the last column. The precise amino acid sequences of the long copep strings identified for all listed vectors are provided in a supplementary table [see Additional file 1]. The E-values listed provide a measure of the specificity with which each corresponding protein is identified by the copep string extracted from a given lsv. Its important to note that the long copep string provides only an approximate summary of the lsv from which it is extracted, yet the small E-values clearly indicate that the vast majority of the proteins identified in Table 2 are very specifically recognized by their corresponding copep string.

**Figure 1:**
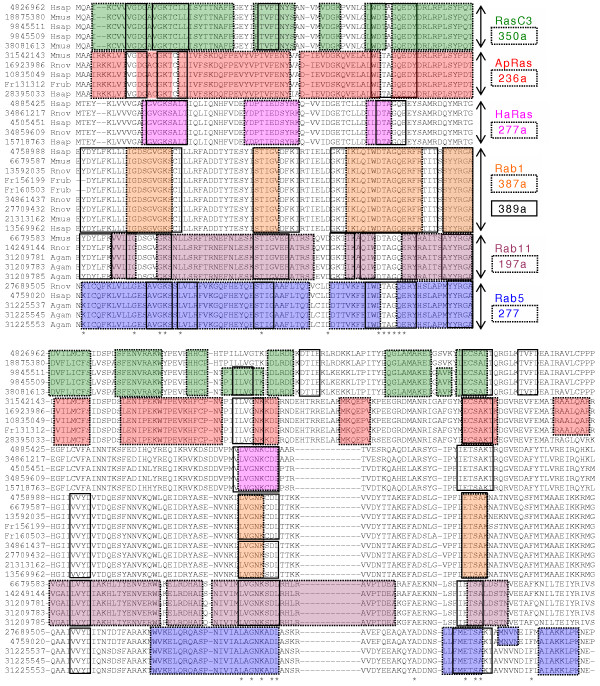
Ras families and sub-families defined by singular vectors (labeled at right). For comparison, dominant peptide strings identified by SVD (boxes) are shown within a Clustal-X alignment. The aligned region corresponds to the first 181aa's of the 192aa Human Rac3 protein. Protein sequences are labeled by gi# (or ensemble# for Frup). Asterisks (*) indicate globally conserved residues. Subfamily motifs associated with negative vector values are denoted with an "a" suffix (e.g. 350a).

Figure 1 provides a more detailed demonstration of how correlated peptide motifs and their associated gene families are simultaneously identified and described by SVD-derived singular vectors. In order to allow a clear comparison of SVD-derived motifs with alignment-derived motifs, the dominant tetrapeptides were superimposed over matching regions of a standard ClustalX alignment of the 34 ras proteins identified in the "top five" of the corresponding right singular vectors listed in Table 3. In this example, the dominant tetrapeptides extracted from the six selected left singular vectors are demarcated within (shaded/colored) boxes. Many of the dominant tetrapeptides are seen to form extended strings of overlapping peptides that correspond well to conserved contiguous regions within particular subsets of the ras proteins. For example, vectors 350a and 236a identify and provide distinct descriptions for motifs within the Ras-related botulinum toxin C3 substrate proteins (RasC3) and the Aplysia-related ras proteins (ApRas), respectively. The two most dominant left singular vectors of Figure 1 (389a and 387a) describe motifs within overlapping subsets of the nine Rab1 proteins. In addition, the most dominant left singular vector (389a) appears to describe a highly conserved motif within the entire set of 34 ras proteins reasonably well (solid clear boxes). This vector conspicuously identifies dominant tetrapeptides that span the two regions of the alignment in which unbroken strings of two or more invariant amino acids (asterisks) are present. These two regions are known to be required for ras GTPase activity [18]. It is notable that although these 34 ras proteins have only one stretch with more than two globally conserved consecutive amino acids (DTAGQE), vector 389a is capable of describing large regions of all 34 proteins by recognizing the latent similarity of multiple equivalent tetrapeptides. For example, this single vector recognizes KSAL, KSCL, and KTCL (residues 18–21 of the alignment) as dominant tetrapeptides that occupy equivalent positions within four of the six subtypes of ras proteins (Figure 1). Vector 389a also provides a reasonably strong summary of the large number of other ras proteins present within the genomes of these organisms, but not included in Figure 1 (not shown). In general, the most dominant singular vectors appear to identify highly conserved peptides present in a high fraction of individual members of a protein family or superfamily, while the less dominant vectors appear to describe conserved tetrapeptides present within a restricted set of proteins comprising a subfamily.

**Figure 2:**
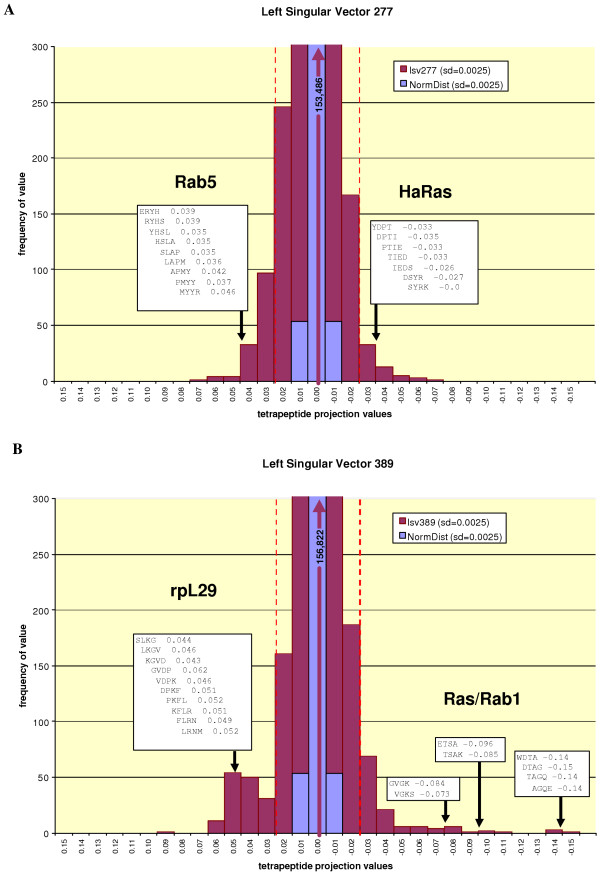
Left singular vectors depicted as tetrapeptide projection value frequency distributions. Distributions for singular vectors 277 (A) and 389 (B) are shown in purple, normal distributions having the same standard deviation are shown in blue. For both distributions, the vast majority of values fall between 0.015 and -0.015. Dashed lines mark the cut-off values used to extract dominant tetrapeptides summarizing correlated peptide (copep) motifs. Selected strings of overlapping tetrapeptides describing parts of these motifs are shown boxed above the approximate regions in the distribution in which they appear.

Instead of simply providing restricted motif summaries using the most dominant elements of the left singular vectors, we have also attempted to examine entire vectors in order to gain a better understanding of the motifs (and associated protein families) they describe. A reasonably efficient method for depicting left singular vectors is presented in Figure 2, using vectors 389 and 277 as examples. Both vectors are shown as frequency distributions (purple bars) that summarize the approximate magnitudes of the projections provided by all 160,000 tetrapeptides on the vector in question. These distributions are compared to a normal distribution having the same standard deviation (blue bars). In both examples, a significant fraction of tetrapeptides have high or low values in considerable excess of that expected from a normal distribution. Many of these also exceed the arbitrary cut-off value of 0.025 (dashed lines) used to extract the dominant tetrapeptides that serve to summarize the corresponding motifs. Parts of the Rab5 and HaRas motifs extracted from vector 277 are shown in Figure 2A as overlapping dominant tetrapeptides with associated projection values. Similar motifs extracted from vector 389 are shown in Figure 2B. In the latter case, a motif from the large subunit ribosomal protein rpL29 represents the "anti-motif"of the Ras/Rab proteins described by the extreme vector elements of opposite sign.

### Species vectors for the production of species phylogenies

**Figure 3:**
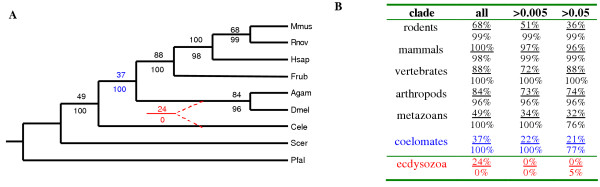
SVD-based proteome phylogeny (A) of nine eukaryotes with percentage branch support: top – bootstrap; bottom – novel jackknife. An unsupported alternative phylogeny containing the "ecdysozoan" lineage is indicated by the dashed red branches. Percentage branch support values for the various clades of the tree are also provided to the left (B) for trees built using all proteins, as well as trees built after poorly described proteins are removed using either of two alternative vector magnitude inclusion values (>0.005, >0.05).

The detailed comparative information contained within the hundreds of singular vectors and their corresponding motifs and gene families was subsequently used to build a species phylogeny by summing all the SVD-derived right protein vectors separately for each organism and then comparing the relative orientation of the resulting species vectors [13]. Figure 3A shows the SVD-based topology obtained for the nine eukaryotes compared in this study. This tree supports a coelomate rather than ecdysozoan lineage. Two distinct re-sampling methods were used to estimate branch statistics for this tree. The top value of each pair of support values for each branch shown in Figure 3A was generated using a traditional bootstrap procedure [19]. In this case, 100 random sets of 437 re-sampled singular vectors were made and used to construct 100 species trees. Alternatively, a novel "successive, delete one" jackknife procedure [14] was used to generate the bottom value shown for each branch. In this case, the least dominant singular vector was removed successively (down to 10 vectors) to generate 427 ordered sets of singular vectors, and a new tree was estimated following each removal. Although bootstrap support values for the branches grouping arthropods with vertebrates (37%) and worms with other metazoa (49%) are relatively weak, support values for these branches are strong (100%) using the modified jackknife procedure. All other branches are strongly supported by both procedures. The branch separating Cele from the coelomates is of special interest, since the weak bootstrap support observed (37%) might suggest a significant affinity between Cele and the arthropods consistent with the "ecdysozoan" model (Figure 3A – alternative branching pattern shown in red). Bootstrap support for the alternative ecdysozoan cluster, however, was only 24%.

Use of the "successive, delete-one" jackknife procedure as a species tree branch statistic is justified by the fact that SVD provides singular triplets in order of their "dominance" in explaining the data set [20]. Mathematical dominance provides an objective measure of importance that can be utilized to weight characters. Since the modified jackknife procedure used here deletes the least dominant singular vectors one at a time in order, the more dominant singular vectors (i.e. conserved motifs/families) are automatically weighted more heavily within the consensus tree. Hence, one can argue that our novel jackknife procedure provides stronger support for the derived phylogeny because the most dominant singular vectors generally contain stronger information about gene and species relationships.

### Poorly described proteins and species tree quality

While our SVD-based analysis technically considers all proteins present within all nine genomes of the data set, it is likely that accurate vector definitions are provided for only a small fraction of these proteins. Theoretically, the 437 singular triplets could effectively describe as many as 2 × 437 = 874 protein families. However, many of these vectors appear to best describe particular subfamilies of larger groups of closely related proteins. Thus, the 58 protein families listed in Table 2 are each represented by anywhere from 1 to 8 triplets. Although, as mentioned earlier, some protein families lacking clear functional annotation were omitted from this table, it still serves to provide a conservative lower estimate of the number of well-described protein families provided by the SVD. Assuming the number of identifiable protein families in our nine genome data set significantly exceeds the 58 to 179 protein families unambiguously demarcated and subdivided in our analysis, then hundreds or perhaps thousand of the poorly described proteins included in our species vector sums might be contributing a high fraction of "noise" to the definition of species.

In an attempt to increase the fraction of well described proteins used to define species, proteins having poor projections on all 437 right singular vectors were ignored during the summation process. Arbitrary vector magnitude cut-off values of 0.005 or 0.05 were applied to reduce the number of poorly described proteins used to build species trees. Even though the highest and most stringent cut-off value removed the majority of proteins during summation, both new species trees had identical topologies to that of the tree shown in Figure 3A in which all proteins were included. Bootstrap and modified jackknife branch support values for these tree are shown in Figure 3B along with those derived from the inclusive analysis. The removal of only a small fraction of poorly described proteins (cut-off = 0.005, about 10^3 ^proteins removed) resulted in 22% bootstrap and 100% modified jackknife support for the coelomate lineage, but 0% support for the ecdysozoan lineage. Removal of a much higher fraction of poorly described proteins (cut-off = .05, about 10^5 ^proteins removed) produced an equivalent result. Hence, poorly described proteins contribute little to the support that our analysis provides for the coelomate model.

## Conclusions

As demonstrated above, an SVD-based analysis of multiple genomes automatically interprets proteins from input genomes as potential members of a limited list of hierarchically defined protein families and subfamilies. Each subfamily is defined in detail by one or more singular vectors as linear combinations of a large number of peptides (160,000 tetrapeptides, in this case). Potentially, a large number of proteomes lacking annotation can be directly interpreted using this method, assuming a sufficient number of annotated proteomes are included in the analysis. Although most of the genomes used in the present analysis were already accompanied by detailed protein annotations, formal annotations of the Frub and Agam proteins were not readily available. Nevertheless, our SVD-based analysis was able to provide precise protein motif descriptions and subfamily affiliations, not only for the six Frub or Agam proteins shown in Figure 1, but also for any of the hundreds of other Frub or Agam proteins exhibiting strong vector projections on any of the 437 derived singular vectors (see "SVD top five" of Table 1).

Our method bears partial resemblance to a recently described graph-theoretic method for rapidly clustering massive datasets of whole genome protein sequence [22]. In this case, the protein definitions generated were not used to derive gene or species trees, but to provide for a comprehensive clustering of all proteins into families having one or more members. The nodes of their graphs, like the vectors from the right matrix in our analysis, represent proteins, while the edges between nodes in their graphs, like the angles between vectors in our analysis, contain the distance information used to compare proteins. However, the distance information in their analysis was obtained ultimately from exhaustive pairwise BLAST alignments. In contrast, our distance information was derived without alignment, by reference to the 437 most dominant SVD-derived orthonormal left singular vectors. These vectors provide "motif models" expressed as particular linear combinations of the 160,000 possible tetrapeptides. The projections of these motif models on a given protein vector serve to quantitatively define the protein. Since no more than 874 motif models would be provided by our truncated SVD, our method would be less effective than other methods for providing comprehensive family designations for all proteins in a dataset [22,23]. However, a high fraction of these protein families are found to contain only one or a few members [22]. Singletons and small families would generally provide unimportant contributions to relative species definitions, since the majority of species would lack a homolog for comparison. Hence small or poorly conserved protein families, presumably represented by the weaker singular triplets in a complete SVD, are profitably ignored in our analysis.

Although our descriptive analysis of singular triplets (e.g. Table 2, Figure 1) suggests that the protein vectors in our high dimensional definition space can be effectively clustered, we have not applied any specific clustering algorithm. Hence no explicit clustering of proteins, equivalent to the identification of orthologs or homologs, is required. Nevertheless, the application of a clustering algorithm to our vector based symmetric protein distances is clearly feasible and results in accurate clustering for a high fraction of proteins. In fact, the accuracy with which proteins are clustered into known families via Neighbor Joining was used previously to establish optimal dimensionality for a well characterized data set [13]. In addition, unlike other methods, our method provides a straightforward vector addition mechanism for converting relative protein definitions into relative species definitions for the production of species phylogenies.

Alternative non-alignment methods exist for comparing sequences [reviewed in [17]]. Some of these methods may prove to be scalable and adaptable to the problem of whole genome phylogeny. For example, a comprehensive bacterial phylogeny was recently derived using species vectors that include a set of background corrected pentapeptide or hexapeptide (K-tuple) frequency values [12]. Although apparently effective for producing global species phylogenies, this method fails to provide quantitatively comparable protein definitions or interpretable predictions for conserved motifs. While many phylogenetically informative pentapeptides and hexapeptides are likely derived from homologs or orthologs, no mechanism exists for extracting, summarizing, and interpreting this information in terms of motif and gene family definitions. This high stringency method provides a low false positive rate (strong connections between probable orthologous peptides), but comes at the expense of a high false negative rate (little or no recognition of other homologous regions within proteins). For organisms exhibiting a significant level of horizontal gene transfer [24-26], models for motifs and protein families may be crucial tools for identifying "borrowed" genes and assessing their impact on phylogenetic hypotheses.

Our SVD-based species tree supports the traditional "coelomate" model of animal phylogeny. Other large-scale, genome level analyses also tend to support this model [27,28]. The alternative "ecdysozoan" model is supported by comparative analyses of rRNA and analyses that include morphological characters [28,29]. Although genome-scale analyses should perhaps carry considerable weight due to the higher fraction of "total information" used as input, the separation of "signal" from "noise" represents a serious hurdle for these methods. Our method represents a uniquely independent solution that provides a noise-reduced simultaneous global comparison of all proteins within multiple genomes without the need for alignments and without the prior application of operational definitions of orthology. As such, it provides a global perspective on gene and species relationships that is based on a much larger subset of information than that normally used. Since it is a non-alignment method, it provides a fundamentally different kind of analysis, and to the extent that the resulting species phylogenies agree with those provided by other analyses that depend upon highly filtered subsets of aligned orthologs or close homologs, we may derive an additional degree of confidence in these relationships. However, the balanced comparison of a large number of additional whole genome sequences from a variety of animals will likely be required in order to produce an unambiguous and universally accepted animal phylogeny.

## Methods

### Datasets

Complete reference protein sequences for nine whole eukaryotic genomes ranging from yeast to man were compiled into a single dataset (Figure 1a). Curated protein sequence files were obtained from NCBI dated as follows: human (Hsap) 10/10/03, mouse (Mmus) 10/31/03, rat (Rnov) 9/23/03, mosquito (Agam) 10/24/03, fly (Dmel) 10/24/03, worm (Cele) 11/12/03, malaria (Pfal) 10/17/02, and yeast (Scer) 11/12/03. Pufferfish (Frub) protein sequences dated 8/26/02 were obtained from the DOE Joint Genome Institute.

### Peptide frequencies and SVD

Each protein sequence in the dataset was recoded as a high dimensional vector containing raw frequencies for each of the 160,000 possible tetrapeptides. Previous work has established that although tripeptides work well for estimating similarities between highly divergent proteins contained within small sets of viral genomes [15], tetrapeptides work better for larger data sets derived from vertebrate mitochondrial genomes or whole bacterial genomes [13,14]. Although pentapeptides also worked well with the mitochondrial datasets (unpublished), our computational capacity precluded the use of pentapeptides (3.2 million patterns) and larger data sets, like the one used here. Following a log-entropy transformation [21], the singular value decomposition of the resulting data matrix was computed. The log-entropy transformation tends to down-weight evenly distributed high frequency peptides that are likely sources of homoplasy. After 1500 Lanczos iterations (residual errors less than 10^-6^), three output matrices were obtained, consisting of 437 singular triplets (left and right singular vectors and their corresponding singular value). Each left singular vector produced by the SVD defines one or two conserved motifs within the dataset as particular linear combinations of tetrapeptides [13,14]. Similarly, each of the right singular vectors defines one or two conserved gene families (or subfamilies) as particular linear combinations of proteins. Each gene family identified by a given right singular vector contains motifs described by the corresponding left singular vector. Two distinct motif/families are frequently identified per triplet, since each triplet describes both a correlated motif/family (positive values) and an anti-correlated motif/family (negative values).

### Vector based motif and protein family models

"Dominant" vector elements (absolute values in excess of 0.025) were extracted from the left singular vectors and summarized using the C++ program "Copepx" [14]. These values were associated with the most "dominant" (i.e. highly conserved) tetrapeptides found within the motifs described by a given left vector. In addition, the "top five" positive and "top five" negative elements were extracted from the right singular vectors and summarized using the C++ program "Coprotx". These values represent the most dominant members of the gene families described by a given right vector.

### Species trees and branch support

Distance matrices were derived by summing all the SVD-derived right protein vectors for a given organism and then comparing the relative orientation of the resulting species vectors using the program Cosdist [13,14]. Species trees were subsequently derived from distance matrices using Phylip-Neighbor [30]. Two distinct resampling methods were used to provide branch support: a traditional bootstrap procedure [19], and a modified jackknife procedure. For the bootstrap, 100 random sets of 437 resampled singular vectors were made and used to construct 100 species trees. For the "successive, delete one" jackknife procedure [14], the least dominant singular vector was removed successively (down to 10 vectors) to generate 427 ordered sets of singular vectors, and a new tree was estimated following each removal.

## List of abbreviations used

Homo Sapiens (Hsap), Mus musculus (Mmus), Rattus Norvegicus (Rnov), Anopheles gambiae (Agam), Drosophila melanogaster (Dmel), Caenorhabditis elegans (Cele), Plasmodium falciparum (Pfal), and Saccharomyces cerevisiae (Scer), Fugu rubripes (Frub), correlated peptide (copep), correlated protein (coprot), right singular vector (rsv), left singular vector (lsv).

## Authors' contributions

GS conceived the study, gathered the input data, provided primary interpretation of the output, and drafted the manuscript. MB wrote and adapted software, performed computational analysis on the input data, and provided manuscript modifications. All authors read and approved the final manuscript.

## Supplementary Material

Additional File 1 Copep Motifs. Long copep strings identified within the left singular vectors of a given s-triplet. Click here for file

## Figures and Tables

**Table 1:** Genes and Genomes Compared

Organism	SVD "top 5"	Genome Total
Hsap	996 (23%)	25,319 (14%)
Mmus	881 (20%)	25,371 (14%)
Rnov	670 (15%)	21,204 (12%)
Frub	536 (12%)	37,439 (21%)
Agam	573 (13%)	16,091 (9%)
Dmel	443 (10%)	18,107 (10%)
Cele	135 (3%)	21,124 (12%)
Scer	113 (3%)	5,855 (3%)
Pfal	23 (1%)	5,049 (3%)
	4370 (100%)	175559 (100%)

**Table 2:** A selected list of protein family/motifs identified by SVD-derived singular triplets (st's). In this summary table, unique example proteins (rsv-gi#) were chosen from the 5 to 40 "top five" proteins identified as members of a given family by as many as 8 distinct right singular vectors. As examples, six individual ras proteins representing six broad categories of ras (highlighted in italics) are defined by a total of 13 right singular vectors, and 18 ribosomal proteins (highlighted in bold) are defined by a total of 65 right singular vectors. The lengths of continuous copep strings identified from the corresponding left singular vectors and their specificities (E-values) as revealed by pairwise BLAST are also provided.

**triplet**	**#**	**rsv-gi#**	**Name**	**Protein Description**	**lsv copep string (E-value)**
421a	1	11415030	HIST1H4J	H4 histone family, member E	62 aa's (1e-54)
417a	2	21166389	HIST1H2BC	H2B histone family, member L	75 aa's (4e-67)
**413a**	**1**	**31560385**	**Rpl21**	**ribosomal protein L21**	60 aa's (2e-55)
408	1	4501885	ACTB	beta actin; beta cytoskeletal actin	42 aa's (9e-38)
**405**	**1**	**4506661**	**Rpl7a**	**ribosomal protein 7a**	79 aa's (3e-62)
392a	1	5174735	TUBB2	tubulin, beta, 2	45 aa's (7e-41)
*389a*	*2*	*13569962*	*RAB1B*	*RAB1B, RAS oncogene family; small GTP-binding*	14 aa's (2e-11)
**389**	**3**	**6677781**	**Rpl29**	**ribosomal protein L29**	77 aa's (3e-60)
387	3	31981690	Hspa8	heat shock 70kD protein 8	40 aa's (2e-35)
385a	1	11024714	UBB	ubiquitin B precursor; polyubiquitin B	77 aa's (2e-68)
378a	5	26051216	CAMK2B	calmodulin-dependent protein kinase IIB isoform 7	14 aa's (2e-10)
373a	2	4502201	ARF1	ADP-ribosylation factor 1	86 aa's (1e-41)
371a	3	6679439	Ppia	peptidylprolyl isomerase A; cyclophilin A	55 aa's (2e-48)
368a	5	25150942	Tcb-1	transposable element tcb1 transposase (1O615)	88 aa's (7e-74)
363	3	33149310	UBE2D3	ubiquitin-conjugating enzyme E2D 3 isoform 1	138 aa's (7e-91)
354	3	4502549	CALM2	calmodulin 2; phosphorylase kinase delta	40 aa's (1e-19)
**352a**	**4**	**17105394**	**RPL23A**	**ribosomal protein L23a**	44 aa's (3e-33)
*350a*	*4*	*9845511*	*RAC1*	*ras-related C3 botox sub 1 isoform Rac1, rho*	15 aa's (2e-12)
347a	3	51873060	Eef1a1	eukaryotic translation elongation factor 1 alpha 1	24 aa's (4e-19)
**345**	**2**	**27679110**	**Rpl17**	**ribosomal protein L17 (L23)**	92 aa's (2e-89)
341a	5	31980772	Ppp1cc	protein phosphatase 1, catalytic, gamma isoform	20 aa's (5e-17)
337	5	24648716	mod(mdg4)	modifier of mdg4	32 aa's (2e-29)
334	5	24653107	Galpha49B	G protein alpha49B	19 aa's (9e-18)
**333a**	**3**	**4506633**	**RPL31**	**ribosomal protein L31**	78 aa's (8e-74)
329a	2	34878793	Pcdha13	protocadherin alpha 13	17 aa's (8e-14)
327	3	32307119	PPP2R2B	Serine/threonine protein phosphatase 2A, neuronal	23 aa's (7e-20)
324	1	31982919	ZNF430	zinc finger protein 430	18 aa's (3e-11)
322a	3	34871376	LOC287293	similar to high mobility group 1 protein	15 aa's (9e-13)
321a	3	4504445	HNRPA1	heterogeneous nuclear ribonucleoprotein A1	23 aa's (2e-18)
320a	2	25141298	kin-1	cyclic AMP-dependent catalytic subunit (kin-1)	66 aa's (4e-62)
316a	5	22094075	Slc25a5	solute carrier family 25; adenine nucleotide	27 aa's (7e-22)
308a	3	9845502	LAMR1	laminin receptor 1 (67kD, ribosomal protein SA)	68 aa's (1e-60)
304	3	6978809	Eno1	enolase 1, alpha	32 aa's (3e-27)
**301**	**4**	**27676004**	**LOC365206**	**similar to ribosomal protein L9**	139 aa's (1e-13)
295	2	31083250	PPP2R5C	Ser/threo protein phosphatase 2A, 56 kD regulator,	16 aa's (6e-12)
**292**	**4**	**31560517**	**Rpl27a**	**ribosomal protein L27a**	58 aa's (7e-56)
**291**	**2**	**15011936**	**RPS26**	**ribosomal protein S26**	77 aa's (7e-64)
288	1	22129671	Olfr493	olfactory receptor MOR204–35	12 aa's (3e-08)
287	2	38076430	LOC193565	similar to T-cell receptor alpha chain	16 aa's (2e-12)
285a	3	6754140	H2-Q7	histocompatibility 2, Q region locus 7	19 aa's (5e-16)
**280a**	**5**	**16418339**	**Rpl10**	**ribosomal protein 10**	27 aa's (4e-23)
*277a*	*1*	*15718763*	*KRAS2*	*cellular c-Ki-ras2 proto-oncogene*	9 aa's (2e-06)
*277*	*2*	*27689505*	*Rab5c*	*similar to Rab5c protein*	17 aa's (4e-13)
276	4	24580529	M(2)21AB	Minute (2) 21AB CG2674-PA	25 aa's (5e-20)
272	1	25742772	Kcna2	potassium voltage-gated channel, shaker-related,	12 aa's (1e-09)
**270**	**4**	**33186863**	**Rpl13**	**ribosomal protein L13**	11 aa's (3e-09)
**266**	**4**	**4506697**	**RPS20**	**ribosomal protein S20**	54 aa's (2e-49)
**256**	**3**	**4506597**	**RPL12**	**ribosomal protein L12**	34 aa's (8e-30)
253a	6	15809016	MRLC2	myosin regulatory light chain MRCL2	19 aa's (7e-16)
**247**	**3**	**31981515**	**Rpl7**	**ribosomal protein L7**	10 aa's (4e-08)
240a	5	24639734	Dlc	dynein light chain ATPase	22 aa's (4e-21)
237a	4	34865959	gpdh	similar to glyceraldehyde-3-phosphate	16 aa's (7e-13)
*236a*	*2*	*10835049*	*ARHA*	*Aplysia ras-related homolog 12; oncogene RHO*	9 aa's (9e-07)
**230**	**6**	**15431293**	**RPL15**	**ribosomal protein L15**	11 aa's (6e-09)
**224**	**5**	**13592069**	**Rps10**	**ribosomal protein S10**	81 aa's (1e-78)
*197a*	*2*	*14249144*	*Rab11b*	*RAB11B, member RAS oncogene family*	15 aa's (4e-12)
**190a**	**6**	**4506621**	**RPL26**	**ribosomal protein L26**	16 aa's (8e-14)
**183a**	**5**	**14277700**	**RPS12**	**ribosomal protein S12**	13 aa's (1e-10)

**Table 3:** Comparison of seven ras family clusters provided by right singular vectors with KOG and Homologen clusters. Only proteins having one of the five strongest projections ("top five") for a given singular vector are used in the comparison. Few genomes have KOG members specifically identified by NCBI, however, most or all of the "top 5" proteins for a given rsv would likely be identified as members of the same KOG family. For 197a (Rab11), the KOG # provided in parentheses is that of the closely related human protein.

**rsv#**	**gi#**	**projection**	**organism**	**GeneName**	**kog#**	**hg#**
197a	6679583	0.06900	Mmus	Rab11b	(0087)	3109
(Rab11)	14249144	0.06892	Rnov	Rab11b	na	3109
	31209781	0.06827	Agam	na	na	3109
	31209783	0.06827	Agam	na	na	3109
	31209785	0.06826	Agam	na	na	3109
236a	31542143	0.05883	Mmus	Arha	na	1257
(ApRas)	16923986	0.05883	Rnov	Arha2	na	1257
	10835049	0.05873	Hsap	RHOA	0393	1257
	28395033	0.05610	Hsap	ARHC	0393	22408
	en131312	0.05412	Frub	na	na	na
277	27689505	0.07229	Rnov	Rab5c	na	20961
(Rab5)	4759020	0.07214	Hsap	RAB5C	0092	20961
	31225537	0.07022	Agam	na	na	20961
	31225545	0.07022	Agam	na	na	20961
	31225553	0.07022	Agam	na	na	20961
277a	15718763	0.04278	Hsap	KRAS2	0395	2159
(HaRas)	4885425	0.04243	Hsap	HRAS	0395	3907
	34861217	0.04243	Rnov	Hras1	na	3907
	4505451	0.04176	Hsap	NRAS	0395	20564
	34859609	0.04165	Rnov	Nras	na	20564
350a	9845511	0.07403	Hsap	RAC1	0393	23126
(RasC3)	38081613	0.07403	Mmus	Rac1	na	23126
	9845509	0.06942	Hsap	RAC1b	0393	23126
	4826962	0.06820	Hsap	RAC3	0393	3705
	18875380	0.06820	Mmus	Rac3	na	3705
387a	34861437	0.03486	Rnov	Rab1B	na	23689
(Rab1)	21313162	0.03413	Mmus	Rab1B	na	23689
	13569962	0.03400	Hsap	RAB1B	0084	23689
	27709432	0.03400	Rnov	Rab1B-like	na	27733
	en156199	0.03396	Frub	na	na	na
389a	4758988	0.04851	Hsap	RAB1A	0084	3067
(Rab/Ras)	6679587	0.04851	Mmus	Rab1A	na	3067
	13569962	0.04840	Hsap	RAB1B	0084	23689
	en160503	0.04824	Frub	na	na	na
	13592035	0.04811	Rnov	Rab1A	na	3067

**Table 4:** Comparison of four unrelated protein clusters provided by right singular vectors with KOG and Homologen clusters. Descriptions for each of these clusters are provided in Table 2. Only proteins having one of the five strongest projections ("top five") for a given singular vector are used in the comparison.

**rsv#**	**gi#**	**projection**	**organism**	**GeneName**	**kog#**	**hg#**
272a	en165011	0.06928	Frub	na	na	na
(Kcna)	25742772	0.06865	Rnov	Kcna2	na	21034
	4826782	0.06834	Hsap	Kcna2	1545	21034
	31543024	0.06821	Mmus	Kcna2	na	21034
	27465523	0.06632	Rnov	Kcna1	na	183
304	12963491	0.101507	Mmus	Eno1	na	1093
(Eno)	6978809	0.101252	Rnov	Eno1	na	1093
	4503571	0.097337	Hsap	Eno1	2670	1093
	51770896	0.092899	Mmus	Eno1	na	1093
	en150208	0.091209	Frub	na	na	na
316a	32189350	0.11376	Rnov	Slc25a5	na	37448
(Slc25)	22094075	0.11343	Mmus	Slc25a5	na	37448
	4502099	0.11202	Hsap	Slc25a5	0749	37448
	en159404	0.1034	Frub	na	na	na
	20863388	0.10117	Mmus	Slc25a4	na	36058
373a	4502201	0.12887	Hsap	Arf1	0070	1253
(Arf)	6680716	0.12887	Mmus	Arf1	na	1253
	11968098	0.12887	Rnov	Arf1	na	1253
	24668762	0.12856	Dmel	Arf79F	0070	1253
	24668773	0.12856	Dmel	Arf79F	0070	1253
