# Supplementary material for: An SVD-based comparison of nine whole eukaryotic genomes supports a coelomate rather than ecdysozoan lineage
Source: BMC Bioinformatics. 2004 Dec 17;5:204. doi: 10.1186/1471-2105-5-204 (PMC544558; doi:10.1186/1471-2105-5-204)
Supplement: Additional File 1 — Copep Motifs. Long copep strings identified within the left singular vectors of a given s-triplet. [file 1471-2105-5-204-S1.doc]

**Additional file 1: Table of long copep strings.** Long copep strings identified within the left singular vectors of a given s-triplet. A corresponding Homologen family designation is also listed for each identified protein obtained from the right singular vector (rsv-gi#).

| **s-triplet** | **rsv-gi#** | **Name** | **hg#** | **lsv-continuous copep string** |
| --- | --- | --- | --- | --- |
| 421a | 11415030 | HIST1H4J | 41460 | GGVKRISGLIYEETRGVLKVFLENVIRDAVTYCEHAKRKTVTAMDVVYALKRQGRTLYGFGG |
| 417a | 21166389 | HIST1H2B | 37800 | EPAKSAPAPKKGSKKAVTKAQKKDGKKRKRSRKESYSVYVYKVLKQVHPDTGISSKAMGIMNSFVNDIFERIASE |
| **413a** | **31560385** | **Rpl21** | **37377** | KGKILAKRINVRIEHIKHSKSRDSFLKRVKENDQKKKEAKEKGTWVQLKRQPAPPREAHF |
| 408 | 4501885 | ACTB | 37442 | LTERGYSFTTTAEREIVRDIKEKLCYVALDFEQEMATAASSS |
| **405** | **4506661** | **Rpl7a** | **31002** | LCRKMGVPYCIIKGKKVAPAPAVVKKQEAKKVVNPLFEKRPKNFGIGQDIQPKRDLTRFVKWPRYIRLQRQRAILYKRL |
| 392a | 5174735 | TUBB2 | 21227 | ESCDCLQGFQLTHSLGGGTGSGMGTLLISKIREEYPDRIMNTFSV |
| *389a* | *13569962* | *RAB1B* | *36154* | LWDTAGQERFRTIT |
| **389** | **6677781** | **Rpl29** | **37382** | IRSYMAKSKNHTTHNQSRKWHRNGIKKPRSQRYESLKGVDPKFLRNMRFAKKHNKKGLKKMQANNAKAPAA 8e-54 |
| 387 | 31981690 | Hspa8 | 38204 | GIDLGTTYSCVGVFQHGKVEIIANDQGNRTTPSYVAFTDT |
| 385a | 11024714 | UBB | 40779 | GGMQIFVKTLTGKTITLEVEPSDTIENVKAKIQDKEGIPPDQQRLIFAGKQLEDGRTLSDYNIQKESTLHLVRGGM |
| 378a | 26051216 | CAMK2B | 7251 | VVHRDLKPENLLL |
| 373a | 4502201 | ARF1 | 1253 | EAREELMRMLAEDELRDAVLLVFANKQDLPNAMNAAEITDKLGEIVTTIPTIGFNVETVEYKNISFTVWDVGGQDKIRPLWRHYY |
| 371a | 6679439 | Ppia | 39843 | RTHTGPGILSMANAGPNTNGSQFFICTAKTEWLDGKHVVFGKVKEGMNIVEAME |
| 368a | 25150942 | tcb-1 | 14500 | RRLQQAGLHGRKPVKKPFISKKNRMARVAWAKAHLRWGRQEWAKHIWSDESKFNLFGSDGNSWVRRPVGSRYSPKYQTEKSVGCKNL |
| 363 | 33149310 | UBE2D3 | 26314 | MALKRINKELSDLARDPPAQCSAGPVGDDMFHWQATIMGPNDSPYQGGVFFLTISKVLLSICLDILRSQWSPALTIHFPTDYPFKPPKVAFTTRIYHPNINSNGSICSLLCDPNPDDPLVPEIARIYKTDRDKYNRI |
| 354 | 4502549 | CALM2 | 37522 | LTDEEDDEEEDEEVDEMIREAFRVFDKDGDGQVNYEEFV |
| **352a** | **17105394** | **RPL23A** | **37379** | RRQPKYPRKSAPRRNKLDHYAIIKFPLTTESAMKKIRTSPTFR |
| *350a* | *9845511* | *RAC1* | 23126 | GQEDYDRLRPLSYPD |
| 347a | 51873060 | Eef1a1 | 31464 | LPLQDVYKIGGIGTVPVGRVETG |
| **345** | **42627879** | **Rpl17** | **13361** | KQWGWTQGRWPKKSAEFLLHMLKNAESNAELKGLDVDSLVIEHIQVNKAPKMRRRTYRAHGRINPYMSSPCHIEMILTEKEQIVPKPEEEVA |
| 341a | 31980772 | Ppp1cc | 36105 | DLICRAHQVVEDGYEFFAK |
| 337 | 24648716 | mod(mdg4) | 45543 | VCSPFFRKMFTQMPSNTHAIVFLNNVSHSALK |
| 334 | 24653107 | Galpha49B | 1566 | MVDVGGQRSERRKWIHCFE |
| **333a** | **4506633** | **RPL31** | **37383** | RSAINEVVTREYTINIHKRIHGVGFKKRAPRALKEIRKFAMKEMGTPDVRIDTRLNKAVWAKGIRNVPYRIRVRLSRK |
| 329a | 40789247 | Pcdha13 | 40694 | LEVNLQNGILFVNSRID |
| 327 | 32307119 | PPP2R2B | 37938 | RGEYNVYSTFQSHEPEFDYLKS |
| 324 | 31982919 | ZNF430 | na | HKRTHTGEKPYKCEECG |
| 322a | 34871376 | HMGb2 | 46092 | EFSKKCSERWKTMS |
| 321a | 4504445 | HNRPA1 | 40723 | ARPHKVDGRVVEPKRAVSREDS |
| 320a | 25141298 | kin-1 | 21942 | AKRVKGRTWTLCGTPEYLAPEIILSKGYNKAVDWWALGVLIYEMAAGYPPFFADQPIQIYEKIVS |
| 316a | 22094075 | Slc25a5 | 37448 | AAGATSLCFVYPLDFARTRLAADVGK |
| 308a | 9845502 | LAMR1 | 37605 | LKFAAATGATPIAGRFTPGTFTNQIQAAFREPRLLVVTDPRADHQPLTEASYVNLPTIALCNTDSPL |
| 304 | 6978809 | Eno1 | 1093 | LPVPAFNVINGGSHAGNKLAMQEFMILPVGA |
| **301** | **27676004** | **Rpl9** | **37328** | KKKRLRVDKWWGNRKELATVRTICSHVQNMIKGVTLGFRYKMRSVYAHFPINVVIQENGSLVEIRNFLGEKYIRRVRMRTGVACSVSQAQKDELILEGNDIELVSNSAALIQQATTVKNKDIRKFLDGIYVSEKGTVQQ |
| 295 | 31083250 | PPP2R5C | 37661 | EILGSIINGFALPLK |
| **292** | **31560517** | **Rpl27a** | **37381** | HHHRINFDKYHPGYFGKVGMRHYHLKRNQSFCPTVNLDKLWTLVSEQTRVNAAKNKTG |
| **291** | **15011936** | **RPS26** | **37420** | GGRGHVQPIRCTNCARCVPKDKAIKKFVIRNIVEAAAVRDISEASVFDAYVLPKLYVKLHYCVSCAIHSKVVRNRS |
| 288 | 22129671 | Olfr | 17188 | STCTSHLTAVT |
| 287 | 38076790 | TcR | 19073 | DSQPGDSATYFCAAS |
| 285a | 6754140 | MHC-H2Q | 37578 | VTLRCWALGFYPADITLT |
| **280a** | **16418339** | **Rpl10** | **38128** | IRTKLQNKEHVIEALRRAKFKFPGRQ |
| *277a* | *15718763* | *KRAS2* | *37990* | YDPTIEDS |
| *277* | *27689505* | *Rab5c* | *37939* | GKSSLVLRFVKGQFHE |
| 276 | 24580529 | MAT | 38112 | TGRKIIVDTYGGWGAHGGGAFSGK |
| 272 | 25742772 | Kcna2 | 21034 | RVFRIFKLSRHS |
| **270** | **33186863** | **Rpl13** | **5568** | LKPHFHKDWQ |
| **266** | **4506697** | **RPS20** | **37417** | KTPCGEGSKTWDRFQMRIHKRLIDLHSPSEIVKQITSISIEPGVEVEVTIADA |
| **256** | **4506597** | **RPL12** | **31003** | IIKALKEPPRDRKKQKNIKHSGNITFDEIVNIA |
| 253a | 15809016 | MRLC2 | 23407 | KLNGTDPEDVIRNAFACF |
| **247** | **31981515** | **Rpl7** | **37375** | EPYIAWGYP |
| 240a | 24639734 | Dlc | 2777 | KYNPTWHCIVGRNFGSYVTHET |
| 237a | 34865959 | gpdh | 46268 | DQVVSCDFNSNSHSS |
| *236a* | *10835049* | *ARHA* | *1257* | VKHFCPNV |
| **230** | **15431293** | **RPL15** | **37713** | GKGHKFHHTIG |
| **224** | **13592069** | **Rps10** | **788** | LFKEGVMVAKKDVHMPKHPELADKNVPNLHVMKAMQSLKSRGYVKEQFAWRHFYWYLTNEGIQYLRDYLHLPPEIVPATL |
| *197a* | *14249144* | *Rab11b* | *37903* | NLLSRFTRNEFNLE |
| **190a** | **4506621** | **RPL26** | **764** | DEVQVVRGHYKGQQIG |
| **183a** | **14277700** | **RPS12** | **36049** | KLGEWVGLCKID |
